# Oxytocin and Vasopressin Are Dysregulated in Williams Syndrome, a Genetic Disorder Affecting Social Behavior

**DOI:** 10.1371/journal.pone.0038513

**Published:** 2012-06-12

**Authors:** Li Dai, C. Sue Carter, Jian Ying, Ursula Bellugi, Hossein Pournajafi-Nazarloo, Julie R. Korenberg

**Affiliations:** 1 Center for Integrated Neuroscience and Human Behavior, and Department of Pediatrics, University of Utah, Salt Lake City, Utah, United States of America; 2 Brain-Body Center, University of Illinois, Illinois, Chicago, United States of America; 3 Department of Medicine, University of Utah, Salt Lake City, Utah, United States of America; 4 Laboratory for Cognitive Neuroscience, Salk Institute, La Jolla, California, United States of America; University of Regensburg, Germany

## Abstract

The molecular and neural mechanisms regulating human social-emotional behaviors are fundamentally important but largely unknown; unraveling these requires a genetic systems neuroscience analysis of human models. Williams Syndrome (WS), a condition caused by deletion of ∼28 genes, is associated with a gregarious personality, strong drive to approach strangers, difficult peer interactions, and attraction to music. WS provides a unique opportunity to identify endogenous human gene-behavior mechanisms. Social neuropeptides including oxytocin (OT) and arginine vasopressin (AVP) regulate reproductive and social behaviors in mammals, and we reasoned that these might mediate the features of WS. Here we established blood levels of OT and AVP in WS and controls at baseline, and at multiple timepoints following a positive emotional intervention (music), and a negative physical stressor (cold). We also related these levels to standardized indices of social behavior. Results revealed significantly higher median levels of OT in WS versus controls at baseline, with a less marked increase in AVP. Further, in WS, OT and AVP increased in response to music and to cold, with greater variability and an amplified peak release compared to controls. In WS, baseline OT but not AVP, was correlated positively with approach, but negatively with adaptive social behaviors. These results indicate that WS deleted genes perturb hypothalamic-pituitary release not only of OT but also of AVP, implicating more complex neuropeptide circuitry for WS features and providing evidence for their roles in endogenous regulation of human social behavior. The data suggest a possible biological basis for amygdalar involvement, for increased anxiety, and for the paradox of increased approach but poor social relationships in WS. They also offer insight for translating genetic and neuroendocrine knowledge into treatments for disorders of social behavior.

## Introduction

Social and emotional responses are so fundamental to human behavior that they are often taken for granted. However the genetic and neurobiological bases of social behavior are largely unknown as are the mechanisms for disruptions in social behavior and emotional regulation that appear throughout the lifespan as features of mental illnesses. These include autism spectrum disorder (ASD), schizophrenia, general social anxiety disorder, post-traumatic stress disorder and depression. Although converging evidence from humans and other mammals suggests that social and emotional behaviors may be regulated by shared neuroendocrine and mesolimbic circuitry [1], [2], [3], closing the gaps between genetic variation and its effect on neuroendocrine and neural functions and between these and behavior requires a multidisciplinary and multisystems approach that captures each of these levels.

The distinct social-emotional phenotypes of Williams Syndrome (WS) make this condition a compelling model to examine the genetic, neuroendocrine and neural systems underlying human sociality. WS is a neurodevelopmental disorder characterized by a strikingly gregarious personality, an increased approach to strangers [4], [5], enhanced emotional reactivity to music [6], [7], but high levels of generalized anxiety [5], [8], poor social judgment and disturbed peer relationships [9], and altered amygdalar responses to fearful and happy faces [10], [11], [12]. In contrast to disorders such as ASD, in which the genetics are complex and largely unknown, WS is unique in that the causal gene deletion (∼28 genes on 7q11.23) associated with the altered behaviors, has been identified. Recent advances in rare cases of small deletions, have shed light on the effects of individual genes, implicating the deletion of *GTF2I and GTF2IRD1* in WS sociability and cognition [13], [14], although the neuroendocrine correlates or mechanisms for these deletions remain unknown. Consequently, WS serves as a unique model for the cross-disciplinary study of human gene-brain-behavior relationships.

Human emotion and social behavior have been linked to neuroendocrine function, in large part through the external administration of hormones. Two hypothalamic neuropeptides, oxytocin (OT) and arginine vasopressin (AVP) regulate reproduction, social behaviors and emotionality in mammals [15], [16], [17], [18]. In humans, exposure to exogenous OT has been related to enhanced trust, emotional empathy, increased and direct eye gaze, to reduced anxiety and reactivity to fearful stimuli, and to parochial altruism [17], [19], [20], [21], [22], [23], [24], [25], [26], [27]. However, the neuropeptide targets and central circuits are unknown and it is unclear whether the sustained levels of intranasal OT recapitulate the endogenous mechanisms of social behavior. Therefore, in contrast to studies of intranasal OT and behavior, we seek to determine whether OT or AVP are involved in reality, in the endogenous determination of baseline levels or in responses to emotional and physical stimuli, using WS as a model. Initial studies of endogenous OT or AVP in human social and emotional behavior suggest correlations of peripheral peptides with behavior in subjects without psychopathology [26], [28] and with disorders of social behavior [29], [30], [31], [32]. However, individual variations are common in studies of endogenous peptides, and whether these engender or are engendered by individual responses to social stimuli is largely unknown [30], [32], [33], [34]. AVP’s behavioral consequences in humans are less well studied, may be more complex and gender specific [35], [36], [37]. Finally, under some, but not all circumstances, the effects of OT and AVP are the same. For example, in pair bond formation [38] and parental behavior [39] there is evidence of similar directional effects of the two peptides, while for behaviors such as aggression [40], anxiety and stress [17], and social approach [41] these peptides appear to have different, and in some cases opposite effect [42]. Based on these data and the distinct WS patterns of social behavior, exaggerated emotional responses, and anxiety, we reasoned that these features might be in part mediated by dysregulated OT and AVP.

Functional and structural imaging has implicated brain circuitry, including the amygdala, in social-emotional behavior in both WS [7], [11], [12] and typical control (TC) subjects [25], [43], although the mechanisms are unknown. For example, in WS, versus TC, there are decreased amygdalar BOLD responses to fearful faces [11], [12] and increased responses to both happy faces [11] and music [7], as well as increased gaze to the eye region [44]. This evidence combines with the established role of OT in regulating amygdalar responses and eye gaze in TC [25], [43] to suggest that increased or dysregulated OT could mediate both in WS.

Music is a potent emotional stimulus. WS subjects show striking interest in and increased emotional and amygdalar responses to music [7], although the effects of music on their social behaviors remain to be studied. The intersection of systems regulating social interactions and responses to music are also poorly understood in typical populations. However, an argument has been made for the role of music and rhythm in entraining social engagement [17], [45], from infancy forward [46]. In addition, similar brain regions are associated with music and emotion regulation [47], [48]. Moreover, several studies tie the neural systems regulated by OT and AVP to social behavior [17] and music [49]. Finally, human genetic analyses have also supported numerous associations of variations in OT and AVP with social function, music and dance [50], [51], [52]. Converging evidence suggests that sociality and responses to music may rely on common, possibly interrelated systems, although definitive experiments are needed. The co-occurrence of social and music features in WS suggests that a common mechanism might contribute to the amygdalar processing of social-emotional stimuli [53] and the positive emotional response to music [54], [55], both in WS and possibly in the typical subjects.

Although the social and emotional stimuli regulating the release of OT and AVP in humans are not well identified, there is some evidence that highly emotional human subjects may release OT in response to aversive stimuli [56]. Stressors also may release AVP, with a variety of effects on behavior [35] and emotion processing [57]. Based on these findings and the high levels of emotional reactivity observed in WS, we also tested OT and AVP responses to a well-characterized negative stressor, cold.

Three independent hypotheses drive the current work. We first hypothesized that basal OT and AVP would be increased in WS, based on the increased initiation of prosocial behaviors in WS (engaging the eyes, approaching both familiars and strangers, expressing empathy and pleasing others). Second, we hypothesized that the increases in basal OT or AVP would be related to social function or dysfunction. We explored this by evaluating the variation of basal OT and AVP and examining the relationship of these with social behaviors, based on both self and observer report [58], [59], [60], [61]. Third, we hypothesized that in WS versus TC subjects, OT and AVP release patterns might show an exaggerated variation and response to either a positive emotional stimulus, music, or to a negative physical, mild stressor, cold.

## Materials and Methods

### Subjects

The WS participants had typical WS deletions determined by fluorescence *in situ* hybridization [62]. The WS cohort included 13 individuals (7 females aged 22–42, and 6 males aged 19–38), previously consented for research studies occurring at the Salk Institute and at Cedars-Sinai Medical Center (CSMC). The current study was approved by the Institutional Review Board at CSMC (IRB Protocol No. 2476), written consent was obtained from participants or their parents/legally authorized representatives and studies were conducted at the CSMC General Clinical Research Center (GCRC). Previous study of the WS group had yielded full scale IQ’s in the range typically observed in WS (46–74) (SOM Table S1). Subjects lived with their parents or in a supported living facility. Written informed consents and assents were obtained from subjects and their parents or legally authorized representative after discussions with Dr. Korenberg. Subjects with mild cognitive impairments were consented or assented according to the process outlined above. Where written consent was obtained from the individual’s parent or legally authorized representative, the individual with WS was read the assent form by research staff in the presence of their representative, and then asked to explain the study in their own words. A research team highly experienced in the measurement of intellectual disabilities, and in assessing the clinical features of WS subjects conducted clinical examinations and evaluated the clinical history of each subject. Typical controls (TC) included 9 subjects (number limited by the IRB) evaluated by physical exam and medical history, frequency-matched to the WS subjects for age-, gender- and ethnicity (4 females aged 20–45, 4 males aged 19–40). Medications recorded (SOM Table S1) for all subjects showed no consistent relationship with OT or AVP levels. Three social behavioral measurements (Adolph’s Approachability, the Salk Institute Sociability Questionnaire (SISQ) and the Scales of Independent Behavior-Revised (SIB-R)) were performed in the WS cohort [58], [59], [60], [61]. Adolph’s Approachability is a self-report measure of an individual’s willingness to approach and speak with a stranger. SISQ and SIB-R are both based on observer reports. SISQ items measure the tendency to approach familiars, strangers and emotional states. SIB-R measures a comprehensive assessment of 14 areas of adaptive behavior and 8 areas of problem behavior. The Salk-McGill Music Inventory Questionnaire of Music Ability and Interest [63] was performed in the WS cohort. All the social behavioral measurements and music questionnaire were performed within 0–3 years of the current study.

### Experimental Paradigm

**Figure 1:**
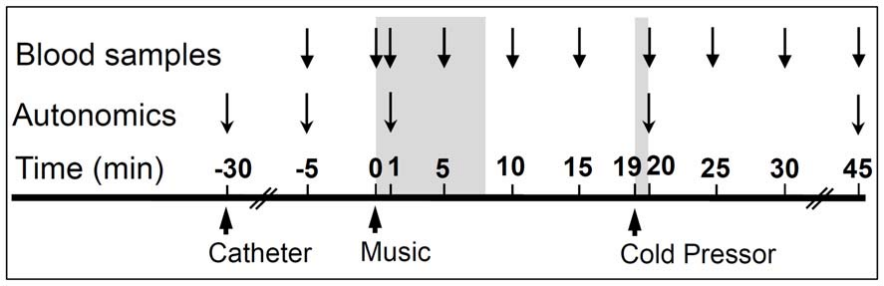
Experimental paradigm used to test neuropeptide and autonomic responses to stimuli in WS and TC. Subjects include 13 WS (7 females, 6 males) and 8 TC (4 females, 4 males). An indwelling catheter was placed 30 mins before blood samples were obtained. Music began at 0 min and continued for 5–8 mins (shaded area); music was selected by each subject as eliciting pleasurable emotional responses. At 19 min, subjects were asked to put one hand in cold water (10–15°C) for 30–60 secs (shaded area). Blood samples were taken prior to (**−**5 and 0 mins), during (1, 5 mins) and immediately following the music (10, 15 mins), and after the cold pressor (20, 25, 30, and 45 mins). The systolic and diastolic blood pressure and heart rate were recorded at **−**30, **−**5, 1, 20 and 45 mins (see Supplemental Information).

The research took place at GCRC at CSMC. Participants were tested one hour after a light meal, between 1–4 P.M. with all samples being drawn between 1–3 P.M. Subjects were instructed to bring their favorite music that elicited positive emotions. This was based on evidence that music described by the individual as intensely pleasurable correlated with fMRI signal in regions implicated in emotion [54] and was the most reliable way to produce intense emotional responses [64]. The experiment was designed as shown in Fig. 1. Participants were tested while supine in a quiet room that was equipped with audio CD facilities. Samples were drawn under conditions that strictly controlled social exposure for the previous 1½ hours. An indwelling catheter was placed by experienced nursing staff, 30 min before testing, during which subjects were alone, lying down in a small room designed and equipped for such human research protocols. To eliminate pain and to minimize the possible effect of anxiety on OT or AVP levels, a lidocaine patch was used prior to insertion of an intravenous catheter, followed by a 1 hour rest period before the first sample was taken. All subjects noted that it did not hurt, and all samples were drawn through the catheter by the same GCRC nurse who had registered them, to minimize the possibility of novel social stimulation. Sensory stimulation was held constant and minimized, except for the music and cold interventions. There were no other subjects in the GCRC and nurses and staff were instructed not to interact with the subject until the experiment was over. Samples were obtained at two baseline points (**−**5 min, 0 min). Music began at 0 min, and continued for 5–8 mins. Multiple, closely spaced blood samples were collected at 1, 5, 10 and 15 min. Intervals between blood samples were selected based on the reported half-life of these neuropeptides [65], [66], [67], which has been estimated at less than 5–10 min. The modified cold pressor stimulus was initiated at 19 min. The subject placed one hand in cold water (15°C) for less than 45 sec, no subject reported pain and all removed the hand at will prior to significant discomfort; blood samples were obtained within 60 secs (at 20 min), and then at 25, 30 and 45 min. Systolic and diastolic blood pressure and heart rate were recorded at **−**30, **−**5, 1, 20 and 45 min.

### Oxytocin/Vasopressin Measurement

Whole blood (5 ml) was collected at each time point in 7 ml purple top tubes containing 5.0 mg EDTA and 2,500 KIU aprotinin (Sigma, USA). Tubes were immediately centrifuged at 1600 g for 5 minutes at 4°C, the sera were aliquoted into 0.5 ml eppendorf tubes, immediately placed on dry ice and stored at **−**80°C. Samples (packed in dry ice and shipped overnight) were sent to University of Illinois at Chicago. Samples did not thaw during shipment and were thawed for the first time immediately prior to assay. The amounts of OT and AVP were determined using enzyme-immunoassays (EIA; Assay Designs/Enzo Life Sciences, Ann Arbor). These assays have been previously validated by multiple methods including parallel experiments with RIA and HPLC as described elsewhere [68]. Standards were run on every EIA plate. All the WS and control samples were run at the same time, on the same equipment, with subsets of high WS and TC samples on the same plate and by a single highly experienced researcher. The EIAs were run in duplicate, on unextracted serum samples that were diluted 1∶4 in dilutent provided by the kits. For the OT and AVP EIA kits, the sensitivity of OT was <11.7 pg/ml and of AVP<3.4 pg/mL with the cross-reactivity between OT and AVP<0.04%. The inter- and intra-assay coefficients of variation were <10% and <11.9% for OT, and <10% and <14.4% for AVP, respectively. Other studies of levels of OT and AVP in typical subjects and clinical populations (schizophrenia and ASD) were performed during the same time period under identical conditions, providing comparison data from several hundred additional subjects, and helping to confirm the validity of these assays [68]. Data from TC subjects in this study were all within the range routinely obtained using these methods. In addition, because data from some WS subjects were outside of the expected range, aliquots of samples from subjects with high levels were rerun and confirmed by serial dilution, generating multiple determinations for each high sample; all of these fell on the linear part of the standard curve, confirming the hormone values presented here.

### Data Analysis

**Figure 2:**
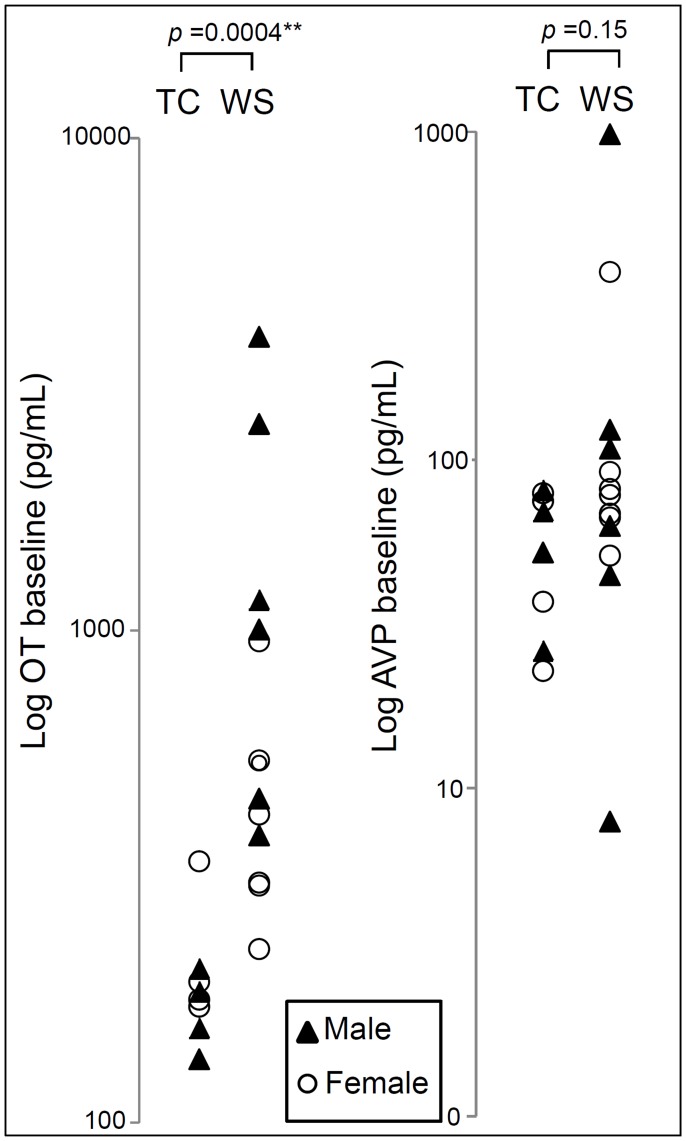
Comparison of basal neuropeptide levels in WS versus TC on the logarithmic scale. WS (n = 13) subjects show 3-fold higher median basal OT (P = 0.0004, determined by the Wilcoxon rank-sum Test) and 1.3-fold higher AVP (P = 0.15) levels versus TC (n = 8). Females are represented by open circles and males by solid triangles.

**Figure 3:**
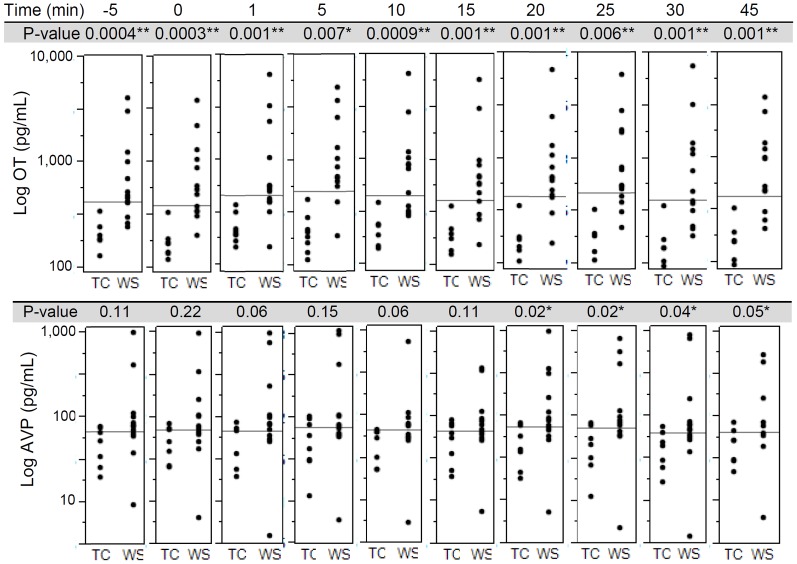
OT levels (pg/mL) measured by enzyme immunoassay in WS cohort are significantly higher than the TC cohort at all ten time points. AVP levels are significantly higher at four out of ten time points. *P<0.05, **P<0.005.

Statistical analyses were performed using SAS and JMP 8. Baseline levels of OT/AVP were defined as the average of measurements at **−**5 and 0 minutes to increase precision. The Wilcoxon rank-sum test, designed to compare independent groups, was used to compare OT and AVP levels between WS and TC at each time point (Fig. 2, 3) because the distributions of both OT and AVP are positively skewed in WS. Spearman’s rho correlation coefficients were applied between the basal levels of OT/AVP and the three social behavioral measurements (Adolph’s Approachability, SISQ and SIB-R). Further, we correlated basal levels of OT and AVP with principal components obtained from the analysis of social behavior measures. The top two components were used; PC1, which loaded most onto adaptive and maladaptive behaviors and PC2, which loaded on to Adolph’s approachability (Table 1). Taking into account that the exact time point of the maximum response was not known in advance, and we hypothesized that the peaked response to occur at somewhat different times for different patients, different music and duration time (chosen by subject to maximize response [54], [69]), we used the peak change (1, 5, 10, 15 mins to music; 20, 25, 30, 45 mins to cold pressor) of response of OT and AVP to the music and cold pressor stimuli. Because we used the same method to calculate peak change in WS and in TCs, the comparison is not biased by the fact that a single time point for peak was not used. The significance in peak change might have been missed had a single peak been used. Statistical methods were designed to remain valid in presence of non-normal data. With our limited sample size, there is insufficient data to perform global comparisons of the overall shape of the curves. Instead, we performed focused comparisons of specific features of the trajectories over time which corresponded to the research hypotheses concerning responses to cold and music. Both raw peak change and peak % change were reported to compare the two groups in response to the stimuli. Given the complex neurobiological systems in humans, we did not expect the response to occur in the same time pattern. The Wilcoxon rank sums test also was used to compare the peak changes between the WS and TC groups, and an exact permutation test was used to compare the variances of the changes between the groups. The permutation test compares the variability in the response between the WS and TC groups and is sensitive to the question of whether there might be a definable subgroup of the WS subjects with greater response to stimuli than other WS subjects. Both the Wilcoxon tests and the permutation tests comparing WS to TC were performed on both the raw and the log transformed scales, as the most appropriate scale for assessing longitudinal change is ambiguous when baseline levels differ [70]. The log transformation reduces the positive skewness for both OT and AVP, observed in the raw data of the WS group, and converts analyses of longitudinal changes to correspond mathematically to analyses of fold change. Based on our original hypothesis that WS versus TC would show increased OT, one-tailed tests were used. A linear regression of baseline OT and AVP on age did not demonstrate a significant effect of age on OT (p = 0.81) or AVP (p = 0.10). No significant differences were found between genders for baseline OT or AVP (t-test; p = 0.11 and 0.51 respectively). We found no evidence of age or gender effects on OT or AVP values and hence did not stratify the statistical analyses by age or gender.

**Table 1:** Basal OT is significantly correlated to Principal Component (PC) 1 (r = **−**0.62, p = 0.04, Spearman’s rho) and PC2 (r = 0.85, p = 0.0008, Spearman’s rho) of three social behavioral measurements in WS cohort (n = 11).

Social behavioral measurements		PC1	PC2
Adolph’s Approachability	Total number of “Yes” responses	0.02	0.44
	Total number of “yes” and “maybe” responses	0.18	0.49
	Sum of all scores	0.19	0.48
SISQ	Total emotionality (expressive and empathy)	**−**0.33	**−**0.01
	Tendency to approach people familiar to the individual	0.19	0.08
	Tendency to approach strangers	**−**0.31	0.05
SIB-R	Motor skills	0.38	0.04
	Social/communication	0.32	**−**0.32
	Personal living	0.38	**−**0.21
	Community living	0.35	**−**0.17
	Internalizing maladaptive	0.23	**−**0.13
	Asocial maladaptive	0.33	0.01
	Externalizing maladaptive	0.15	0.37

PC1 accounts for 39% of the variance and PC2 for 24%. Eigenvectors are shown in this table.

## Results

WS patients were characterized genetically and behaviorally, and in the current study, their neuropeptide responses were compared to age-, gender-, and ethnicity-matched TC.

### OT and AVP Levels are Higher in WS vs TC at Baseline and after Stimuli

The median basal OT level was 3-fold higher in WS versus TC (WS median = 538 pg/mL, TC median = 181 pg/mL, P<0.001) and the median AVP level was 1.3-fold higher in WS (WS median = 78 pg/mL, TC median = 61 pg/mL, P = 0.15 using the Wilcoxon rank-sum test). Basal OT and AVP were positively skewed when expressed as raw data, but were approximately symmetric when shown on a logarithmic scale (Fig. 2). A surprisingly low intra- individual variation was observed in each of the controls over the 10 time points (80 samples total), analyzed at the same time as the WS samples. Previous studies of large numbers of other subjects would not have detected this stability of OT and AVP in that they refer primarily to baseline measures or single subjects; the specific repeated measures design used here is relatively unique to this study. Nonetheless, the single measures from other large studies increased our confidence that the exceptionally high OT and AVP values in our WS subjects were not typical of the population at large and unlikely to have occurred by chance. WS OT levels are higher than TC (p = <0.007) at all ten time points. The AVP levels are increased in WS versus TC, show a trend at baseline and from time 1 to 15 min, but differ significantly at all other time points from 20 to 45 min. (Fig. 3). The slightly smaller size of the control group is accounted for in the p-values and confidence intervals; it reduced our statistical power, but did not compromise the validity of our inferences. The higher basal OT and AVP in WS were also compared to an age and gender-matched subset of TC run in the same time period [71], previously measured by the same method (for OT, TC median = 255 pg/mL, P = 0.001; for AVP, TC median = 57 pg/mL, P = 0.05).

### Correlation of Basal OT and AVP to Social Behaviors in WS

We further investigated how the basal OT/AVP are related to social behaviors in WS, by correlating with three social behavioral measurements, Adolphs Approachability [58], [59], SISQ [61] and SIB-R [60]. A principal components analysis (Table 1) indicated that approach behavior measured by Adolph’s Approachability was the major loading factor for PC2, which correlated significantly with basal OT (r = 0.85; P = 0.0008). In contrast, adaptive social and maladaptive behaviors loaded highly onto PC1, which was significantly negatively correlated with basal OT levels (r = **−**0.62, P = 0.04). The relationship of decreased adaptive social behaviors to increased basal OT was supported by negative correlations between basal OT and specific social subtests (social communications of the SIB-R; r = **−**0.79, P = 0.0035; community living, r = **−**0.67, P = 0.02). Basal OT was not correlated with FSIQ in WS (Spearman’s rho, p = 0.36). Similarly, the adaptive behavior social communications subtest was not correlated with FSIQ (p = 0.41). Community living was correlated with FSIQ (p  = 0.04) but not after regression of OT (p = 0.13). No significant correlations were found between AVP and these adaptive social behavioral measures in WS.

### Responses of OT and AVP to Music and of OT to Cold, are Greater and More Variable in WS than TC (Figs. 3, 4, 5, and 6)

**Figure 4:**
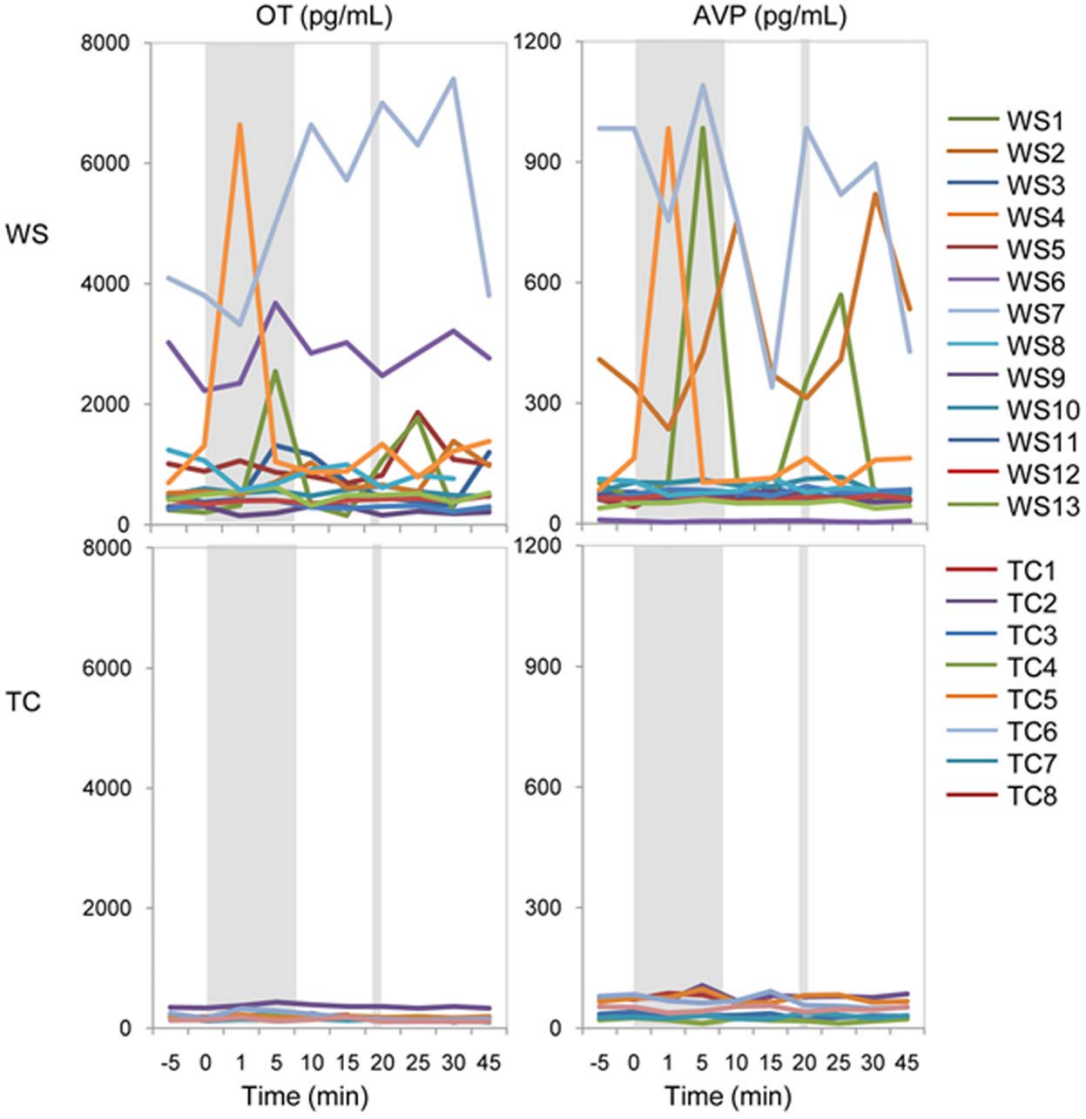
Differences in longitudinal OT and AVP levels for the combined WS versus TC cohorts shown on a common scale without the logarithmic transformation. WS (upper) and TC (lower) panels; OT (left) and AVP (right). The shaded areas represent the duration of music and cold pressor stimuli described in Fig. 1.

**Figure 5:**
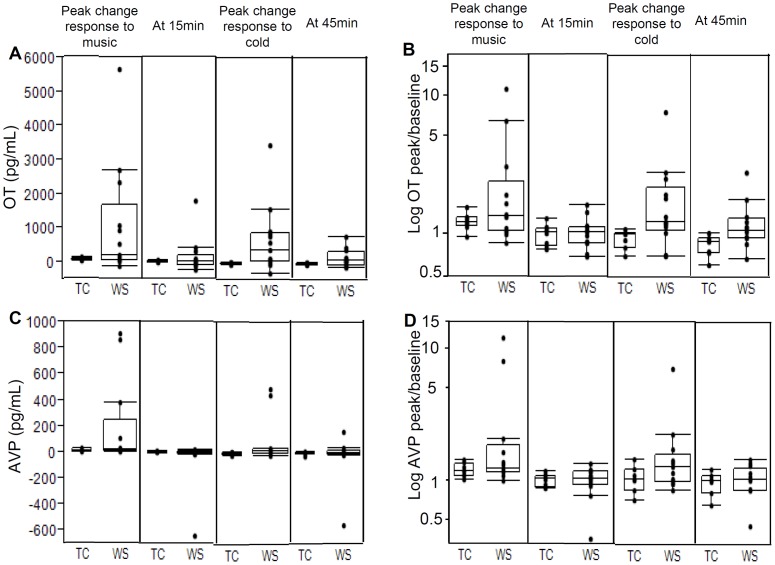
Responses of OT and AVP to music and of OT to cold, are greater and more variable in WS than in TC. Differences in longitudinal OT (upper, A, B) and AVP (lower, C, D) levels for the combined WS versus TC cohorts response to music and cold are shown on a raw scale without the logarithmic transformation on the left (A, C) and ratio versus baseline with the logarithmic transformation on the right (B, D).

**Figure 6:**
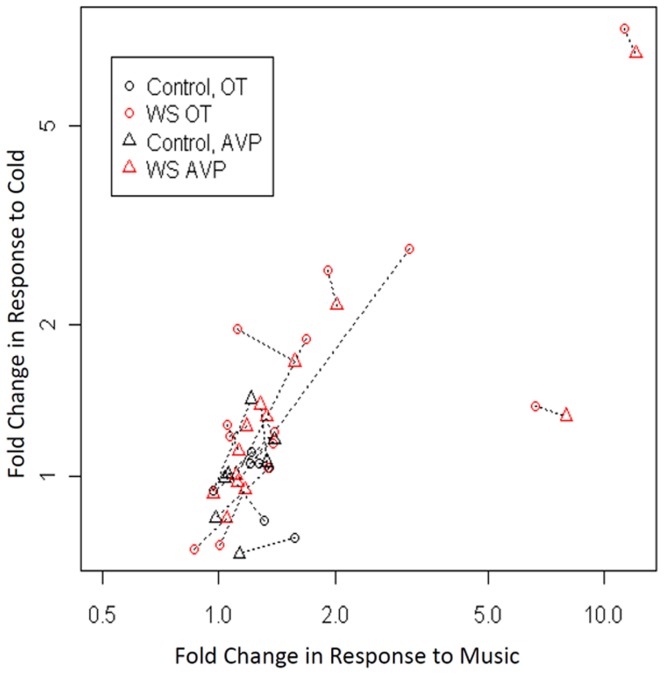
Correlations of peak responses to music and cold for OT and AVP show different patterns in WS versus TC. Shown are the maximum fold-changes in response to cold (vertical axis) and in response to music (horizontal axis) for OT (circle) and AVP (triangle), with dashed lines drawn between the plot symbols for maximum fold changes in OT and AVP corresponding to the same patient. The plot exhibits positive correlations, in WS but not in the TC group, between fold changes in both OT (R = **−**0.25, P = 0.59 for the TC, and R = 0.77, P = 0.004 for WS patients) and AVP (R = 0.57, P = 0.18 for the TC and R = 0.84, P<0.0001 for WS patients) between the two stimuli. A total of 14 fold changes for either OT or AVP exceeded 1.8, all occurring within 6 WS patients.

The statistical methods used here were designed to remain valid in the presence of non-normal data. With our limited sample size, there are insufficient data to perform global comparisons of the overall shape of the curves. Instead, we performed focused comparisons of specific features of the trajectories over time which corresponded to the research hypotheses concerning responses to cold and music. In sensitivity analyses we found that change scores calculated using samples immediately prior to exposure to music were similar to those calculated using the baseline average. We used the average for our primary analyses presented here to improve precision and minimize artifacts of measurement and state. Longitudinal raw measures (Fig. 3) of OT and AVP are significantly greater at all 10 time points and show striking responses to both stimuli (Fig. 4) in WS than in TC, as shown below. The longitudinal changes in OT (expressed as % Δ versus baseline in the peak response during and immediately following the stimulus) exhibited both greater variability (P = 0.025 with music; P = 0.007 with cold pressor, using permutation test) and greater increases in WS versus TC (38% versus 24% median increase, P = 0.21, with music; 26% versus 4% increase, P = 0.01) (Table 2, Fig. 5). The longitudinal changes in AVP exhibited similar but weaker trends, which did not reach statistical significance, for greater variability and greater average increases in WS versus TC (Table 2, Fig. 5). In contrast to WS, in TC, only small increases were seen at 1 min with minimal variation within the cohort. The OT returned towards baseline levels at 15 min (P = 0.44) after the peak response to music. The simultaneously greater variability and greater average increases in WS versus TC, reflect a pattern, displayed in Fig. 6, in which the 14 largest fold changes of >1.8 in either OT or AVP in response to either stimulus all occurred within a subset of 6 patients, all of whom belonged to the WS group. These findings support the distinctness of responses in WS, but also reveal marked individual variation in both baseline peptides and in response to stimulation. Finally, there is a positive correlation in WS, but not TC, between the endocrine responses to music and cold for both OT (R = **−**0.25, P = 0.59 for the TC, and R = 0.77, P = 0.004 for WS patients) and AVP (R = 0.57, P = 0.18 for the TC and R = 0.84, P<0.0001 for WS patients) when measured as % change (Fig. 6). In addition, there are positive correlations in WS, but not TC, between OT and AVP responses to music (R = **−**0.1, P = 0.82 for TC, and R = 0.55, P = 0.015 for WS) and a trend in the WS response to the cold pressor (R = 0.32, P = 0.48 for the TC, and R = 0.48, P = 0.094 for WS). The variation in WS is striking with respect to that in TC.

**Table 2:** Analyses of longitudinal changes in OT and AVP based on raw and log transformed data. *P<0.05, **P<0.005.

Longitudinal Outcome Domain	Scale of analysis (raw change or % change)	Median (mean), TC		Median (mean), WS		Comparison of median changes between groups, *p*-value		Interquartile range (SD), TC		Interquartile range (SD), WS		Comparison of variances of changes between groups, *p*-value	
		OT	AVP	OT	AVP	OT	AVP	OT	AVP	OT	AVP	OT	AVP
Response to Music	Peak change, raw scale	40.1 (51.3)	8.1 (10.6)	173.0 (1027.6)	16.9 (181.9)	0.06	0.045*	44.8 (38.6)	17.0 (11.0)	1011.9 (1653.0)	97.3 (326.9)	0.001**	0.09
	Peak % change	24 (25)	14 (16)	38 (160)	18 (161)	0.21	0.19	16 (18)	23 (14)	85 (303)	46 (340)	0.025*	0.11
15 minutes	Change, raw scale	10.9 (2.3)	0.74 (0.36)	20.7 (151.1)	0.7 (**−**48.8)	0.40	0.43	50.1 (29.6)	6.3 (5.6)	138.3 (515.6)	14.3 (179.0)	0.005**	0.08
	% change	4.4 (1.3)	0.5 (**−**2.1)	5.4 (4.8)	0.7 (**−**2.5)	0.46	0.33	26.5 (17.9)	17.0 (10.3)	24.7 (27.0)	19.7 (23.5)	0.25	0.13
Response to Cold	Peak change, raw scale	6.5 (**−**4.3)	0.5 (**−**0.2)	381.4 (644.4)	13.9 (80.9)	0.01*	0.08	44.1 (26.0)	17.6 (12.6)	774.5 (985.2)	36.0 (172.1)	<0.001**	0.07
	Peak % change	4 (**−**3)	1 (3)	26 (98)	26 (68)	0.01*	0.12	24 (14)	36 (24)	80 (188)	42 (163)	0.007*	0.25
45 minutes	Change, raw scale	**−**16.2 (**−**24.3)	**−**0.2 (**−**4.2)	64.1 (140.9)	**−**0.3 (**−**29.9)	0.051*	0.36	39.3 (24.7)	12.4 (11.9)	296.9 (267.6)	18.4 (172.0)	<0.001**	0.16
	% change	**−**9.1(**−**14.2)	**−**1 (**−**5.0)	9.4 (26.9)	**−**0.6 (0.5)	0.009*	0.30	21.3 (15.2)	26.9 (18.0)	34.3 (57.2)	36.0 (26.6)	0.81	0.20

### Correlation of Basal and Peak Changes of OT and AVP in WS to Self-reported Response to Music

No significant correlations were found between OT or AVP levels and 7 items of a music questionnaire (The Salk-McGill Music Inventory Questionnaire of Music Ability ad Interest [64]). However, increased OT fold change in response to music tended to be related to self-reported time spent on music-related activities (r = 0.57, P = 0.06, Spearman’s rho, not corrected).

### Autonomic Measurements

The systolic and diastolic blood pressure and heart rate are greater in WS than in TC at all time points (P value from 0.02 to 0.55; SOM Fig. S1).

## Discussion

The results of this study provide the first evidence that OT and AVP are both dysregulated in WS. Specifically, basal OT and to a lesser extent AVP, are elevated in WS versus TC, and are related to measures of WS social behavior. Moreover, results indicate that emotional (music) and physically aversive (cold) stimuli cause an exaggerated release of OT and AVP (to music and trend to cold) in people with WS, independent of their basal levels. With respect to WS social behavior, as hypothesized, higher levels of basal OT were correlated with increased approach to strangers but unexpectedly, also to decreased adaptive social behaviors. These results support our hypothesis that in WS, the neurobiological mechanisms that underly intensified emotional responses to music and possibly social behavior, may in part involve the dysregulated synthesis or release of both OT and AVP from the hypothalamic-neurohypophyseal system. Finally, the results indicate that subset(s) of the ∼28 WS deleted genes and their altered expression ultimately disturb the mechanisms underlying the development or adult regulation of OT and AVP-related brain structures and consequently insight into their role in human emotion.

Our data indicate a paradigm shift toward understanding OT as an endogenous modulator of human behaviors that may not always be adaptive in daily life; similar conclusions have been suggested by recent reports of the effects of intranasal OT [21], [72], [73], [74], [75], [76], [77]. The results show striking correlations between basal OT and the standardized measures of social behavior including indices of “approachability” and “sociability” in WS [58], [59], [60], suggesting that elevated OT might “dose-dependently” predispose WS subjects to atypical motivation for social engagement [78], [79]. Moreover, the PC correlations further support the inverse relationship of OT to both increased approach and decreased maladaptive behavior. Nonreciprocal and excessive social behaviors, when combined with intellectual disability in WS, also contribute to their atypical social relationships [80]. Individuals with WS can become perseverative and lack reciprocal reactions. The high levels of OT and correlated drive to approach to strangers, combined with poor judgment, inappropriate perseverance and deficiency in adaptive behaviors, may together predispose patients with WS to their observed decreases in adaptive and increases in maladaptive social interactions. Future studies are necessary to determine the role of basal and induced OT release in WS responses to social interactions. In other social disorders such as ASD, there is also evidence for dysregulated levels of OT [31], [81], although in ASD these have not yet been correlated with individual differences in behavioral scales. We note that although WS and ASD, are at opposite ends of the spectrum of social approach dysfunction, both are associated with poor social outcomes, suggesting the possibility that the behavioral consequences of OT in a normal range may be more advantageous in social interaction and that levels at the high or low end may predispose to disorders such as WS and ASD.

Our data show clearly that WS deleted genes ultimately alter the hypothalamic-pituitary release and/or regulatory mechanisms affecting of OT and AVP, and in turn, the response to emotional and physical stimuli reported here, but it is unclear as to which brain regions may also be involved. These conclusions rest on the well established observation that peripheral levels of OT are determined almost exclusively by hypothalamic-pituitary release [82], and that in rodents [83], increased peripheral levels in response to emotional stimuli, usually but not invariably reflect intracerebral levels. For example, in rats, stress-induced increases in central OT but not AVP, appeared and were correlated with the periphery [84], [85], [86] suggesting that, if similar in humans, increased blood levels of OT in WS subjects might reflect a parallel central release. Therefore, our findings combine with the literature to establish that in WS, the hypothalamic and pituitary release of OT and AVP is dysregulated, and that it is likely but not certain, that central release from the hypothalamus is also affected although the specific brain regions remain to be defined. Similar to the administration of intranasal OT, the data herein inform neither the site of action of these peptides nor the neural circuit that underlies the behavior, which is especially obvious in a subset of WS subjects. Finally, in addition to central effects, circulating peptides may have indirect effects on brain or behavior through actions on brainstem and autonomic pathways that are outside the blood brain barrier [87]. In conclusion, although current methodologies do not permit a noninvasive analysis of the relationship between typical or atypical peripheral neuroendocrine patterns in WS, and their role in specific brain regions remain to be systematically examined, our data clearly establish that OT and AVP are involved in the WS endogenous response circuitry for human emotion, music and physical stress.

Nonetheless, with the caveats above, our findings combine with recent work [43], [88] employing intranasal OT and fMRI, to suggest not-unreasonal hypotheses indicating the neural networks that may modulate social behavior in WS. These may include changes in brain regions implicated in social behavior, eg., orbital frontal cortex, amygdala [11], [12], and insula [89]. Specifically, individuals with WS show an atypical amygdalar BOLD response to emotional faces, decreased to fearful and increased to happy faces [11]. To explain this, it has been proposed that fronto-amygdalar circuitry is altered in WS [12] or that amygdalar development is abnormal [11]. However, our OT data in WS suggest possible alternative explanations. That is, if intracerebral release were also elevated, OT and AVP acting on amygdalar OT or V1a receptors, may predispose to the observed decrease in reactivity to fearful and increase to happy faces, to the prolonged face and direct eye gaze [90], and to the difficulty disengaging from facial versus object stimuli [91] seen in WS. Support for these hypotheses comes from recent observations showing that the effects of intranasally-infused OT [43] on amygdalar BOLD responses in normals, are parallel to WS (without exogenous OT) and differentiate effects on anterior (happy, sad) versus posterior (attention shift to eyes). With respect to the effects of elevated AVP release in WS, emerging data support its role in emotional responses to facial stimuli [92], [93], suggesting a similar role in WS. We hypothesize that increased and exaggerated release of neuropeptides including OT and AVP, may affect specific amygdalar regions and contribute to the increased eye gaze, attention to faces, and social behavior including the inappropriate tendency to approach strangers. The neural circuit connecting hypothalamic release with amygdalar function [94] is an important question for the future.

Music is a universal stimulus for human mood induction and can in some cases release OT [49]. Our finding that listening to music amplified the release of OT and AVP, suggests that dysregulation of both OT and AVP may be related to the exaggerated emotional response to music often reported in WS [95]. A trend in OT fold change which was related to self-reported time spent on music warrants further study of social engagement or music as a means of coping with anxiety in WS. Studies of subjective emotional responses to evocative stimuli may provide more direct evidence to link the OT/AVP response to emotional reactivity. The neuropeptide response to music observed here does not exclude a broader sensitivity to external stimulation in several modalities, which could include hyperacusis [96] and tactile sensitivity, both of which have also been reported in WS. The results implicate OT and for the first time, AVP, in the emotional response to music and provide a physiological rationale for its use as an adjunct in the treatment of disorders of social behavior.

We note that the individual variation in OT baselines and response to both music and cold appears to distinguish a subgroup of WS with particularly exaggerated responses, as shown in Figs. 3, 4, 5, and 6. This variation in basal and dysregulated OT is similar to that seen for many phenotypic features of WS and other genetic disorders. That is, the WS deletion causes a subset of diagnostic features, each of which is present in 5–90% of individuals [97]. Therefore, it is significant that, despite this variation, the effect sizes of exaggerated responses of OT between WS and TC are highly significant, even in the presence of behavioral variation within the WS cohort. Moreover, the correlations observed here with social approach and adaptive behaviors are not limited to those at the highest fold changes but involve the entire WS cohort. Factors influencing the variable synthesis or release of OT and its relationship to social behaviors and emotion in WS will be of interest to determine, and include allelic and non-allelic modifiers, epigenetic effects and pre- and post-natal environment. The results implicate a biological basis for WS emotion and behavior, particularly their eye gaze and attention to faces [22], [27].

The next frontier for understanding the role of OT and AVP in the endogenous brain response, in contrast to its ability to exogenously alter social behavior, will involve their response to specific social stimuli such as emotional faces in WS. The first goals were to establish baselines under highly controlled conditions and to optimize the detection of dysregulation of the OT and AVP system in WS. From previous studies, music had been defined as a consistently strong inducer of emotion in toddlers through adults with WS whereas differential response to emotional faces in WS versus TC is more subtle, thus far, only detected in an regions-of-interest covering the amygdala, and will require further preliminary work to identify a set of facial stimuli that more consistently evoke responses across a cohort of WS. Therefore, this must be viewed as the first in a series of investigations of peptide responses to emotional and social stimuli in WS and the control population. The results now provide the basis on which to formulate future studies of social behavior t hat drill deeper into the neural and neuroendocrine circuitry of social responses through integration of emotional and social stimuli, neural imaging, and neuropeptide response.

It is important to note that the increases in peptide values in WS are ultimately due to the deletion of the ∼28 genes in WS region. However, it is unknown at present where these atypical values are due to direct regulation by WS genes or other indirect genetic or behavioral pathways. Genetic variation in the OT or AVP receptors may also contribute to peptide levels. The coincident elevation of both peptides in WS suggests perturbation of a process in WS that is common to OT and AVP, possibly related to disturbed common autoregulatory release mechanism [98], [99]. This may be due to functional alterations or altered signaling, increased synthesis, trafficking, secretion, defective degradation or epigenetic effects on peptide receptors, such as those reported in ASD [100]. Whether the relationships between OT and behavioral styles reported here also are moderated by interactions between OT and AVP or their known binding to the AVPR1A receptor, needs further study. It is also possible that the increased OT and AVP seen in some patients with WS may be part of a more general exaggerated neuropeptide response to a number of stimuli that may nonetheless affect social or emotional responses. These provide preliminary evidence for a possible common genetic regulatory mechanism for increasing both OT and AVP, whose differential effect on behavior may nonetheless be determined by eg., gender or species specific distribution of their receptors. Future studies are warranted to examine consequences of OT and AVP in humans, which may extend well beyond the social context.

We also observed a decreasing trend in systolic blood pressure in response to music in WS but not TC (SOM Fig. S1). Although autonomic differences between WS and TC did not reach statistical significance, this offers tentative support for the hypothesis that disruptions in the autonomic nervous system are associated with the emotional reactivity of some patients with WS [101]. However, the lack of significant correlation between autonomics and neuropeptide levels at baseline or changes after stimuli suggests a more complex mechanism.

Neuropeptides have not been previously measured in WS, and the current data suggest that endogenous neural circuitry involving OT and AVP may contribute to the exaggerated response to emotional stimuli seen in some individuals with WS, detected here through the measurement of peripheral peptides. Although, as shown here, the hypothalamic-pituitatry axis is clearly involved, the central neuroendocrine and genetic circuits regulating OT and AVP are not well identified, especially in nonreproductive states [102]. However, these may be informed by the current results. Finally, our report of baseline increases and experimentally induced amplification of OT and/or AVP, as well as our previous data linking GTF2I and GTF2IRD1 in WS social behavior [13], implicates a role for these genes as well as others in the WS deleted region including *STX1A*, *LIMK1 and CYLN2,* in the developmental and/or regulatory pathways that determine OT and AVP levels, and consequently, in the human social-emotional behaviors seen in WS [4], [13], [103]. Future studies will be needed to confirm our findings in WS and to link these to specific patterns of disturbed social behaviors as seen in autism spectrum disorder and other genetic and environmental variations that distinguish the normal spectrum of human behavior.

## Supporting Information

Figure S1 **Shown are the mean systolic (A) and diastolic (B) blood pressure and heart rate (C) in WS and TC at all time points, including baseline (−30 and −5 min), 1, 20 and 45 min.** There is a decreasing trend (not reaching statistical significance between WS and TC) in systolic blood pressure and heart rate response to music in WS but not TC.
(TIF)

Table S1 **Demographic characteristics and medications for WS subjects (A) and typical controls (B) in this study.**
(DOCX)
