# Supplementary figures and images for: Oxytocin and Vasopressin Are Dysregulated in Williams Syndrome, a Genetic Disorder Affecting Social Behavior
Source: PLoS One. 2012 Jun 12;7(6):e38513. doi: 10.1371/journal.pone.0038513 (PMC3373592; doi:10.1371/journal.pone.0038513)

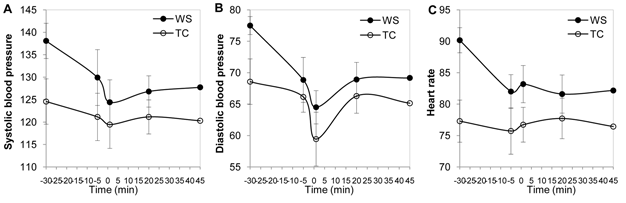

Supplement: Figure S1 — Shown are the mean systolic (A) and diastolic (B) blood pressure and heart rate (C) in WS and TC at all time points, including baseline (−30 and −5 min), 1, 20 and 45 min. There is a decreasing trend (not reaching statistical significance between WS and TC) in systolic blood pressure and heart rate response to music in WS but not TC. (TIF) [file pone.0038513.s001.tif]
