# Supplementary material for: Oxytocin and Vasopressin Are Dysregulated in Williams Syndrome, a Genetic Disorder Affecting Social Behavior
Source: PLoS One. 2012 Jun 12;7(6):e38513. doi: 10.1371/journal.pone.0038513 (PMC3373592; doi:10.1371/journal.pone.0038513)
Supplement: Table S1 — Demographic characteristics and medications for WS subjects (A) and typical controls (B) in this study. (DOCX) [file pone.0038513.s002.docx]

**Supplemental Table S1.** Demographic characteristics and medications for WS subjects (A) and typical controls (B) in this study.

A.

| Williams syndrome | Gender | Age (y/o) | Ethnicity | WAIS-R  FSIQ | Medications |
| --- | --- | --- | --- | --- | --- |
| WS1 | MALE | 32 | Caucasian | 63 | None |
| WS2 | FEMALE | 22 | Caucasian | 46 | BCP |
| WS3 | FEMALE | 31 | Caucasian | 67 | Lexapro, Vasotensin |
| WS4 | FEMALE | 42 | Caucasian | 65 | BCP |
| WS5 | MALE | 19 | Caucasian | 63 | Atenolol, Levothyroxine, Ritalin |
| WS6 | MALE | 38 | Caucasian | 50 | Atenolol |
| WS7 | FEMALE | 26 | Caucasian | 74 | Effexor, Strattera, Klonopin, Trazodone |
| WS8 | MALE | 31 | Caucasian | 72 | None |
| WS9 | FEMALE | 35 | Caucasian | 64 | Abilify, Paxil, Risperdal, Protonix, Clonidine |
| WS10 | FEMALE | 38 | Caucasian | 77 | Zocor, Vytorin |
| WS11 | FEMALE | 40 | Caucasian | 72 | BCP, Toprol |
| WS12 | MALE | 19 | Caucasian |  | None |
| WS13 | MALE | 19 | Hispanic | 64 | None |

B.

| Normal controls | Gender | Age (y/o) | Ethnicity | Medications |
| --- | --- | --- | --- | --- |
| TC1 | FEMALE | 28 | Caucasian | None |
| TC2 | FEMALE | 45 | Caucasian | None |
| TC3 | FEMALE | 20 | Caucasian-Hispanic | None |
| TC4 | FEMALE | 31 | Caucasian | None |
| TC5 | MALE | 20 | Black-Filipino | None |
| TC6 | MALE | 30 | Caucasian | Wellbutrin, Prevacid, Adderall |
| TC7 | MALE | 21 | Caucasian | N/A |
| TC8 | MALE | 40 | Caucasian | B12 |
